# Isocaloric intake of a high-fat diet modifies adiposity and lipid handling in a sex dependent manner in rats

**DOI:** 10.1186/1476-511X-10-52

**Published:** 2011-04-12

**Authors:** Maria E Estrany, Ana M Proenza, Isabel Lladó, Magdalena Gianotti

**Affiliations:** 1 Grup de Metabolisme Energètic i Nutrició, Departament de Biologia Fonamental i Ciències de la Salut, Institut Universitari d'Investigació en Ciències de la Salut (IUNICS), Universitat de les Illes Balears, Cra. Valldemossa Km 7.5. E-07122 Palma de Mallorca, Spain; 2 Ciber Fisiopatología Obesidad y Nutrición (CB06/03), Instituto de Salud Carlos III. Spain

## Abstract

**Background:**

High-fat (HF) diet feeding usually leads to hyperphagia and body weight gain, but macronutrient proportions in the diet can modulate energy intake and fat deposition. The mechanisms of fat accumulation and mobilization may differ significantly between depots, and gender can also influence these differences.

**Aim:**

To investigate, in rats of both sexes, the effect of an isocaloric intake of a diet with an unbalanced proportion of macronutrients on fatty acid composition of visceral and subcutaneous adipose tissues and how this is influenced by both dietary fatty acids and levels of proteins involved in tissue lipid handling.

**Methods:**

Eight-week-old Wistar rats of both sexes were fed a control diet (3% *w/w *fat) or high-fat diet (30% *w/w *fat) for 14 weeks. Fatty acid composition was analyzed by gas-chromatography and levels of LPL, HSL, α2-AR, β3-AR, PKA and CPT1 were determined by Western blot.

**Results:**

The HF diet did not induce hyperphagia or body weight gain, but promoted an increase of adiposity index only in male rats. HF diet produced an increase of the proportion of MUFA and a decrease in that of PUFA in both adipose depots and in both sexes. The levels of proteins involved in the adrenergic control of the lipolytic pathway increased in the gonadal fat of HF females, whereas LPL levels increased in the inguinal fat of HF males and decreased in that of females.

**Conclusion:**

Sexual dimorphism in adiposity index reflects a differential sex response to dietary fatty acid content and could be related to the levels of the proteins involved in tissue lipid management.

## Introduction

Adipose tissue has traditionally been considered the primary site for whole body energy storage, but is also a metabolically active tissue with endocrine functions that secretes a variety of cytokines and hormones [1]. Distinct fat regions have different metabolic activities and biological functions, and an excess of intra-abdominal fat may have a greater impact on lipid and glucose metabolism and on cardiovascular health than subcutaneous fat [2-4].

The amount of fat deposited at any specific adipose tissue site reflects the balance between rates of lipolysis, fatty acid uptake and lipogenesis [5]. These processes may differ between depots and depends on the regulation of several enzymes. LPL is the rate-limiting enzyme for the mobilization of lipid from plasma lipoprotein, determining triacylglycerol accumulation in adipose tissue [6]. HSL, the enzyme involved in lipolytic activity, is activated by hormones such as catecholamines, and its action depends on the balance between beta and alpha receptors in specific adipose tissue sites [7]. Carnitine palmitoyltransferase 1 (CPT1) plays a key role in lipid oxidation through the transport of long chain fatty acids across mitochondrial membranes [8]. The variations in tissue fatty acid uptake and lipolytic activity between fat depots have been shown to be sex dependent and could be factors determining whether excess fat is stored subcutaneously or intra-abdominally [9-12].

Diet composition is known to influence energy intake and body weight changes in rats and humans [13]. High-fat (HF) diets cause an imbalance of energy homeostasis leading to an increase in body fat deposition and are often associated with several morbidities, such as cardiovascular diseases or type 2 diabetes [14]. There is evidence suggesting that not only the quantity but also the quality of dietary fat may influence adipose tissue composition [15]. The proportions in which fatty acids appear in adipose tissue may have a significant importance in the pathophysiology of this tissue [16]. Moreover, fatty acids are recognized as important metabolic effectors that regulate gene expression of the proteins involved in lipid and carbohydrate metabolism [17,18].

Taking into account that body composition and fat distribution are sex dependent and are also affected by nutrient proportion in diet, the aim of the present study was to determine whether the macronutrient composition of the diet affects the profile of fatty acid composition of rat gonadal and inguinal adipose tissues in a sex dependent manner. We further analyzed the influence of both dietary fatty acids and levels of proteins involved in tissue fatty acid uptake and release on adiposity in both sexes.

## Materials and methods

### Materials

Gas liquid chromatography internal (heptadecanoic acid) and external (fatty acid methyl ester mixture) standards and capillary column SP-2330 were from Sigma-Aldrich (St Louis, MO, USA). Rabbit antibody against PKA and goat antibodies against α2- and β3-AR were obtained from Santa Cruz Biotechnology (Santa Cruz, CA, USA). Rabbit antibody for CPT1 was obtained from Alpha Diagnostic International (San Antonio, Texas, USA). Rabbit antibody for HSL was kindly provided by Dr. F.B. Kraemer and chicken antibody for LPL was previously developed in our laboratory [19]. Enhanced chemiluminescence western blotting analysis reagents were supplied by Amersham (Little Chalfont, UK). The kit for serum cholesterol measurement was purchased from Linear Chemicals SL (Barcelona, Spain). Accutrend^® ^GCT-meter and triacylglyceride and glucose test strips were purchased from Roche Diagnostics (Basel, Switzerland). Non-esterified fatty acid assay kit was obtained from Wako Chemicals (Osaka, Japan). Routine chemicals were purchased from Sigma-Aldrich (St Louis, MO, USA) and Panreac (Barcelona, Spain). Both control and high-fat diets were obtained from Panlab (Barcelona, Spain).

### Animals and diets

Animal experiments were performed in accordance with general guidelines approved by our institutional ethics committee and EU regulations (86/609/CEE and 2003/65/CE). Eight-week-old Wistar rats (Charles River, Barcelona, Spain), 14 males and 14 females, were housed two per cage with free access to food and water in a temperature controlled room (22°C) under a 12 h light/dark cycle. For each sex, rats were divided into 2 experimental groups: the control group (rats fed a pelleted control diet) and the high-fat (HF) group (rats fed a pelleted high-fat diet). The content of protein, carbohydrate and fat in the control diet represented 18.7%, 73.3% and 8.0%, respectively, of the total energy content of the diet. In the HF diet, protein, carbohydrate and fat were 13.5%, 31.3% and 55.2%, respectively, of the total energy content of the diet. Diet composition is shown in Table 1.

**Table 1:** Nutrient composition of diets

	Control diet		HF diet	
Fat	2.90		30.0	
C14:0	nd		0.53	(1.77)
C16:0	0.47	(16.2)	7.70	(25.7)
C18:0	0.13	(4.48)	5.30	(17.7)
C16:1n-7	0.04	(1.38)	1.10	(3.67)
C18:1n-9	0.65	(22.4)	13.3	(44.3)
C18:2n-6	1.40	(48.3)	3.60	(12.0)
C18:3n-3	0.12	(4.14)	nd	
SFA	0.60	(20.7)	13.0	(43.3)
MUFA	0.70	(24.1)	14.0	(46.7)
PUFA	1.50	(51.7)	3.00	(10.0)
ΣSFA/ΣMUFA	0.86		0.93	
ΣSFA/ΣPUFA	0.40		4.33	
Protein	15.5		16.6	
Carbohydrate	60.5		20.6	
Fiber	3.90		3.60	
Mineral mix	5.30		3.50	
Vitamin mix	1.00		0.80	
Energy content	1410		2106	

Nutrient composition and energy content of the diets are given in g/100 g and KJ/100 g of diet, respectively. The percentage of each fatty acid with respect to the total amount of fat of the diet is indicated in parentheses. SFA, saturated fatty acids; MUFA, monounsaturated fatty acids; PUFA, polyunsaturated fatty acids. nd: not detected.

Energy intake and body weight were determined weekly and fortnightly, respectively, throughout the 14 weeks of the experiment. Rats were sacrificed by decapitation after a 12-hour period of fasting. Gonadal and inguinal fat depots were removed, weighed, immediately frozen in liquid nitrogen and stored at -80°C until analyzed.

### Adipose tissue composition and fatty acid analysis

Adipose tissue samples were homogenized with a Teflon/glass homogenizer in Tris sucrose buffer (5 mM Tris-HCl, 0.25 mM sucrose, 2 mM EGTA, pH 7.4) and protein and DNA content were determined [20,21]. Aliquots of homogenates were used for western blot analysis. Lipids were extracted from adipose tissue samples by the method of Folch et al. [22]. Fatty acids in the extract were determined as previously described [23,24]. The relative amount of a given fatty acid was expressed as the percentage of its integrated chromatogram peak area with respect to the total area of fatty acids that were identified (C14:0, C16:0, C18:0, C20:0, C16:1n-7, C18:1n-9, C18:2n-6, C18:3n-3). SFA was defined as the sum of C14:0, C16:0, C18:0 and C20:0. MUFA was defined as the sum of C16:1n-7 and C18:1n-9. PUFA was defined as the sum of C18:2n-6 and C18:3n-3. Owing to their low levels, not all fatty acids were quantifiable in all samples.

### Western blot analysis of adipose tissue LPL, HSL, α2-AR, β3-AR, PKA and CPT1 protein levels

50 μg (CPT1), 40 μg (LPL, HSL) or 25 μg (α 2-AR, β3-AR, PKA) of protein were fractionated on 8-10% SDS-PAGE gels and electrotransferred onto nitrocellulose filters. Membranes were incubated overnight at 4°C (HSL, α2- AR, β3- AR, PKA, CPT1) or for 1 hour at room temperature (LPL) in blocking solution. Development of the immunoblots was performed using an enhanced chemiluminescence kit. Bands were visualized with the ChemiDoc XRS System (Bio-Rad, Hercules, CA, USA) and analyzed with the image analysis program Quantity One (Bio-Rad, Hercules, CA, USA). Bands revealed an apparent molecular mass of 60, 84, 55, 58, 42 and 88 KDa for LPL, HSL, α2-AR, β3-AR, PKA and CPT1, respectively.

### Statistical analysis

The results are presented as mean values ± SEM of 7 animals per group. Statistical tests were performed using a statistical software package (SPSS version 17 for windows, Inc., Chicago, IL, USA). Statistical differences between experimental groups were analyzed by two-way analysis of variance (ANOVA). Student's t-test was performed as post-hoc comparison when an interactive effect of sex and diet was shown. A p < 0.05 was considered statistically significant. Pearson's correlations coefficients were used to analyze the association between tissue fatty acid composition and adiposity index or dietary fatty acids.

## Results

### Food, energy and macronutrient intake

Food intake of HF animals was lower than that of controls, but due to the high caloric content of the HF diet, the energy intake was slightly higher than controls (Table 2). Food, energy and protein and lipid intake were higher in female rats than in males. In both sexes, the consumption of HF diet reduced the intake of carbohydrates (75% of decrease) and proteins (20% of decrease) and augmented that of lipids (eight fold increase). The intake of individual fatty acids was also significantly increased in HF animals compared to controls, mainly that of SFA and MUFA (fifteen fold increase), whereas the consumption of PUFA was increased almost twice.

**Table 2:** Daily food, energy and macronutrient intake

	Male		Female		
			
	Control	HF	Control	HF	ANOVA
Food intake (g/kg BW^0.75^)	42.6 ± 0.9	31.1 ± 0.8*^a^*	44.9 ± 1.4	33.7 ± 0.8*^a, b^*	S, D
Energy intake (kJ/kg BW^0.75^)	605 ± 11	663 ± 18*^a^*	634 ± 21	716 ± 15*^a, b^*	S, D
Carbohydrate intake (g/kg BW^0.75^)	25.8 ± 0.5	6.41 ± 0.17*^a^*	27.2 ± 0.8	6.94 ± 0.16*^a^*	D
Protein intake (g/kg BW^0.75^)	6.56 ± 0.13	5.16 ± 0.14*^a^*	6.92 ± 0.21	5.59 ± 0.13*^a, b^*	S, D
Lipids intake (g/kg BW^0.75^)	1.23 ± 0.03	9.24 ± 0.25*^a^*	1.30 ± 0.04	10.0 ± 0.2*^a, b^*	S, D
C14:0 (mg/kg BW^0.75^)	-	165 ± 4	-	179 ± 4	-
C16:0 (mg/kg BW^0.75^)	200 ± 4	2394 ± 65*^a^*	211 ± 6	2594 ± 61*^a, b^*	S, D, SxD
C18:0 (mg/kg BW^0.75^)	55.4 ± 1.1	1648 ± 45*^a^*	58.4 ± 1.8	1786 ± 42*^a, b^*	S, D, SxD
C16:1n-7 (mg/kg BW^0.75^)	17.0 ± 0.3	342 ± 9*^a^*	17.9 ± 0.6	371 ± 9*^a, b^*	S, D, SxD
C18:1n-9 (mg/kg BW^0.75^)	277 ± 6	4136 ± 112*^a^*	292 ± 9	4481 ± 105*^a, b^*	S, D, SxD
C18:2n-6 (mg/kg BW^0.75^)	596 ± 12	1120 ± 31*^a^*	629 ± 19	1213 ± 28*^a, b^*	S, D
C18:3n-3 (mg/kg BW^0.75^)	53.9 ± 1.7	-	51.1 ± 1.0	-	-
SFA (mg/kg BW^0.75^)	256 ± 5	4043 ± 110*^a^*	266 ± 9	4379 ± 102*^a, b^*	S, D, SxD
MUFA (mg/kg BW^0.75^)	299 ± 6	4354 ± 118*^a^*	314 ± 10	4717 ± 110*^a, b^*	S, D, SxD
PUFA (mg/kg BW^0.75^)	639 ± 13	933 ± 25*^a^*	674 ± 21	1011 ± 24*^a, b^*	S, D

Values are expressed as the mean ± S.E.M. of 7 animals per group. BW, body weight. ANOVA (p < 0.05): S, sex effect; D, diet effect; SxD, interactive effect; Student's t-test (p < 0.05): *^a^*HF *vs *Control, *^b^*Female *vs *Male.

### Serum lipid profile and glucose

The consumption of the HF diet decreased serum triacylglycerols in both sexes and free fatty acid levels only in male rats (Table 3). Glucose and cholesterol levels were lower in female rats than in males. Glucose levels were not modified by HF feeding.

**Table 3:** Serum lipid profile and glucose

	Male		Female		
			
	Control	HF	Control	HF	ANOVA
Triacylglycerols	2.48 ± 0.16	2.08 ± 0.15	2.90 ± 0.41	2.05 ± 0.27	D
Free fatty acids	0.906 ± 0.043	0.665 ± 0.037*^a^*	0.928 ± 0.081	0.948 ± 0.046*^b^*	S, D, SxD
Total cholesterol	1.74 ± 0.12	1.63 ± 0.15	1.50 ± 0.10	1.26 ± 0.14*^b^*	S
Glucose	8.20 ± 0.27	8.10 ± 0.60	6.62 ± 0.28*^b^*	7.00 ± 0.32	S

Values are expressed in mM and are the mean ± S.E.M. of 7 animals per group. ANOVA (p < 0.05): S, sex effect; D, diet effect; SxD, interactive effect; Student's t-test (p < 0.05): *^a^*HF *vs *Control, *^b^*Female *vs *Male.

### Weight and composition of adipose tissue depots

Consumption of HF diet did not modify body weight in either sex (Table 4). In response to HF diet, only male rats increased fat depots and adiposity.

**Table 4:** Body weight, adiposity index and adipose tissue composition

	Male		Female		
			
	Control	HF	Control	HF	ANOVA
Body weight (g)	452 ± 9	470 ± 13	257 ± 8*^b^*	263 ± 3*^b^*	S
Adiposity index (%)	5.55 ± 0.57	9.16 ± 0.67*^a^*	5.78 ± 0.67	6.40 ± 0.71*^b^*	D, SxD
Gonadal fat					
Weight (g/kg BW)	16.8 ± 2.0	28.8 ± 2.4*^a^*	18.7 ± 2.8	24.5 ± 3.4	D
DNA (mg/g)	19.4 ± 2.3	12.5 ± 0.9*^a^*	10.8 ± 1.2*^b^*	11.8 ± 0.5	S, D, SxD
Protein (mg/g)	4.33 ± 0.39	3.79 ± 0.16	5.28 ± 0.41	4.03 ± 0.38*^a^*	D
(mg/mg DNA)	0.231 ± 0.025	0.311 ± 0.024*^a^*	0.497 ± 0.033*^b^*	0.342 ± 0.029*^a^*	S, SxD
Lipids (mg/g)	144 ± 11	174 ± 17	176 ± 15	130 ± 12*^a, b^*	SxD
(mg/mg DNA)	8.31 ± 1.36	13.4 ± 1.2*^a^*	14.6 ± 1.9*^b^*	10.6 ± 1.2	SxD
Inguinal fat					
Weight (g/kg BW)	17.1 ± 1.7	24.8 ± 2.5*^a^*	15.7 ± 1.7	17.1 ± 2.0*^b^*	S, D
DNA (mg/g)	7.70 ± 1.45	8.87 ± 0.87	11.4 ± 2.59	7.73 ± 0.49	NS
Protein (mg/g)	9.71 ± 1.86	5.67 ± 0.42*^a^*	15.0 ± 2.2	8.73 ± 1.15*^a, b^*	S, D
(mg/mg DNA)	1.15 ± 0.27	0.669 ± 0.087*^a^*	1.39 ± 0.14	1.14 ± 0.12*^b^*	S, D
Lipids (mg/g)	158 ± 10	103 ± 21*^a^*	138 ± 8	159 ± 10*^b^*	SxD
(mg/mg DNA)	15.4 ± 2.2	22.3 ± 5.3	14.0 ± 5.3	18.0 ± 2.5	NS

Adiposity index was calculated as the sum of the weight of lumbar, gonadal, inguinal and mesenteric fat depots expressed as a percentage with respect to body weight. Values are expressed as the mean ± S.E.M. of 7 animals per group. BW, body weight. ANOVA (p < 0.05): S, sex effect; D, diet effect; SxD, interactive effect; NS, non significant; Student's t-test (p < 0.05): *^a^*HF *vs *Control, *^b^*Female *vs *Male.

Protein content was lower in HF rats than in controls with the exception of gonadal fat of males when expressed per cell. In inguinal depot, protein content was higher in female rats than in males but no effects of sex or diet were observed in DNA content. DNA levels of gonadal fat were lower in female rats compared to males and decreased only in males in response to dietary treatment. HF diet brought about a decrease of lipid content only in gonadal fat of female rats.

### Fatty acid composition of adipose tissue depots

In both fat depots of control rats, C16:0 was the main fatty acid followed in percentage by C18:1n-9 and C18:2n-6 (Table 5). In contrast, in HF animals the main fatty acid of both gonadal and inguinal tissues was C18:1n-9 followed in percentage by C16:0 and C18:2n-6. With the HF diet consumption, MUFA increased and PUFA decreased in both adipose tissues of both sexes, while SFA increased in the inguinal depot but did not change in the gonadal one. Accordingly, in both fat depots the SFA to MUFA ratio was lower while the SFA to PUFA ratio was higher in HF rats of both sexes. In both adipose tissues, dietary treatment increased C18:0 and C18:1n-9 proportions and decreased those of C18:2n-6. Sex effects were mostly observed in inguinal fat. Thus, MUFA content was higher in female rats than in males (due mainly to the proportion of C18:1n-9); the PUFA content of control females was lower than that of males and decreased to similar values in both sexes with the dietary treatment; the increased C18:0 content induced by HF diet feeding was higher in female rats than in males.

**Table 5:** Fatty acid composition of gonadal and inguinal adipose tissue depots

	Male		Female		
			
	Control	HF	Control	HF	ANOVA
Gonadal fat					
C14:0	2.63 ± 0.15	1.90 ± 0.07*^a^*	2.35 ± 0.16	2.85 ± 1.18*^b^*	S, SxD
C16:0	30.9 ± 1.4	30.8 ± 0.4	33.7 ± 0.4*^b^*	33.0 ± 1.2	S
C18:0	4.24 ± 0.59	5.74 ± 0.22*^a^*	3.8 ± 0.3	5.2 ± 0.6*^a^*	D
C20:0	1.16 ± 0.19	0.442 ± 0.021*^a^*	1.04 ± 0.02	0.481 ± 0.064*^a^*	D
C16:1n-7	6.30 ± 0.51	3.27 ± 0.07*^a^*	5.6 ± 0.5	4.4 ± 0.6	D
C18:1n-9	28.9 ± 3.2	46.4 ± 0.4*^a^*	30.6 ± 0.8	39.2 ± 2.5*^a, b^*	D, SxD
C18:2n-6	23.5 ± 2.4	10.7 ± 0.2*^a^*	22.4 ± 0.3	12.2 ± 1.3*^a^*	D
C18:3n-3	0.247 ± 0.032	0.267 ± 0.018	0.177 ± 0.013	0.230 ± 0.040	NS
SFA	39.1 ± 1.5	39.0 ± 0.4	41.3 ± 1.3	41.7 ± 1.3*^b^*	S
MUFA	35.3 ± 3.4	49.9 ± 0.4*^a^*	36.3 ± 0.7	43.8 ± 2.6*^a^*	D
PUFA	23.7 ± 2.4	11.0 ± 0.2*^a^*	22.6 ± 0.3	12.4 ± 1.2*^a^*	D
ΣSFA/ΣMUFA	1.18 ± 0.16	0.782 ± 0.014*^a^*	1.14 ± 0.04	0.978 ± 0.093	D
ΣSFA/ΣPUFA	1.76 ± 0.22	3.56 ± 0.09*^a^*	1.83 ± 0.04	3.56 ± 0.42*^a^*	D
Inguinal fat					
C14:0	2.90 ± 0.44	2.39 ± 0.11	2.62 ± 0.14	2.61 ± 0.17	NS
C16:0	30.5 ± 0.9	31.9 ± 0.7	32.0 ± 0.4	29.9 ± 0.5*^a, b^*	SxD
C18:0	3.03 ± 0.16	5.77 ± 0.16*^a^*	2.96 ± 0.22	7.04 ± 0.19*^a, b^*	S, D, SxD
C20:0	1.22 ± 0.03	0.431 ± 0.035*^a^*	1.02 ± 0.03*^b^*	0.387 ± 0.015*^a^*	S, D, SxD
C16:1n-7	6.68 ± 0.59	3.68 ± 0.22*^a^*	6.69 ± 0.43	3.72 ± 0.21*^a^*	D
C18:1n-9	27.7 ± 0.8	42.1 ± 0.9*^a^*	30.7 ± 0.8*^b^*	44.4 ± 0.6*^a, b^*	S, D
C18:2n-6	27.8 ± 0.6	10.9 ± 0.5*^a^*	23.8 ± 0.4*^b^*	10.9 ± 0.2*^a^*	S, D, SxD
C18:3n-3	0.307 ± 0.075	0.264 ± 0.085	0.165 ± 0.011	0.345 ± 0.054	NS
SFA	38.0 ± 1.3	40.8 ± 0.8*^a^*	39.0 ± 0.6	40.3 ± 0.7	D
MUFA	34.5 ± 0.5	46.0 ± 1.1*^a^*	37.5 ± 1.0*^b^*	48.4 ± 0.6*^a, b^*	S, D
PUFA	28.1 ± 0.6	11.2 ± 0.5*^a^*	23.9 ± 0.4*^b^*	11.3 ± 0.2*^a^*	S, D, SxD
ΣSFA/ΣMUFA	1.11 ± 0.05	0.891 ± 0.034*^a^*	1.05 ± 0.05	0.833 ± 0.024*^a^*	D
ΣSFA/ΣPUFA	1.36 ± 0.07	3.72 ± 0.23*^a^*	1.63 ± 0.04	3.58 ± 0.10*^a^*	D

Fatty acid content was expressed as a percentage of its integrated chromatogram peak area with respect to the total area of fatty acids that were identified (C14:0, C16:0, C18:0, C20:0, C16:1n-7, C18:1n-9, C18:2n-6, C18:3n-3). Values are expressed as the mean ± S.E.M. of 7 animals per group. ANOVA (p < 0.05): S, sex effect; D, diet effect; SxD, interactive effect; NS, non significant; Student's t-test (p < 0.05): *^a^*HF *vs *Control, *^b^*Female *vs *Male.

Fatty acid desaturation indexes (16:1/16:0 and 18:1/18:0) decreased in response to HF diet feeding in both sexes and in both depots (Table 6), except the 18:1/18:0 ratio which did not change in HF animals.

**Table 6:** Lipid desaturation indexes in gonadal and inguinal adipose tissue depots

	Male		Female		
			
	Control	HF	Control	HF	ANOVA
Gonadal fat					
16:1/16:0	0.207 ± 0.020	0.106 ± 0.002*^a^*	0.171 ± 0.012	0.135 ± 0.020	D, SxD
18:1/18:0	7.49 ± 1.10	8.14 ± 0.29	8.66 ± 0.56	8.14 ± 1.02	NS
Inguinal fat					
16:1/16:0	0.217 ± 0.015	0.115 ± 0.007*^a^*	0.181 ± 0.032	0.111 ± 0.015*^a^*	D
18:1/18:0	9.25 ± 0.49	7.34 ± 0.30*^a^*	9.24 ± 1.74	5.68 ± 0.69*^a, b^*	D, SxD

Values are expressed as the mean ± S.E.M. of 7 animals per group. ANOVA (p < 0.05): D, diet effect; SxD, interactive effect; NS, non significant; Student's t-test (p < 0.05): *^a^*HF *vs *Control, *^b^*Female *vs *Male.

### Levels of proteins involved in adipose tissue lipid uptake and mobilization

In gonadal fat, HSL, α2-AR and β3-AR levels were higher in control female rats than in males (Table 7). In this depot, HF diet feeding induced an increase of β3-AR and PKA levels and a decrease of CPT1 content and α2-AR/β3-AR ratio in both sexes; α2-AR levels decreased in HF female rats. LPL levels did not change between sexes or in response to dietary treatment.

**Table 7:** Levels of proteins involved in adipose tissue lipid uptake and mobilization

	Male		Female		
			
	Control	HF	Control	HF	ANOVA
Gonadal fat					
CPT1	100 ± 18	55.2 ± 2.1*^a^*	83.4 ± 8.0	64.6 ± 12	D
LPL	100 ± 8	89.8 ± 13.8	71.4 ± 10.6	84.4 ± 12.1	NS
HSL	100 ± 14	58.4 ± 11.0	135 ± 17	154 ± 32*^b^*	S
PKA	100 ± 8	131 ± 17	90.9 ± 8.2	131 ± 16*^a^*	D
α2-AR	100 ± 7	103 ± 4	132 ± 7*^b^*	99.8 ± 12.8*^a^*	SxD
β3-AR	100 ± 12	127 ± 8*^a^*	137 ± 9*^b^*	155 ± 5*^b^*	S, D
α2/β3- AR	1.05 ± 0.11	0.82 ± 0.05*^a^*	0.97 ± 0.06	0.64 ± 0.06*^a^*	D
Inguinal fat					
CPT1	100 ± 23	34.8 ± 7.6*^a^*	39.6 ± 11.4*^b^*	24.2 ± 7.6	S, D
LPL	100 ± 23	344 ± 35*^a^*	204 ± 36*^b^*	133 ± 36*^b^*	D, SxD
HSL	100 ± 37	88.8 ± 32.0	97.0 ± 15.4	98.7 ± 37.6	NS
PKA	100 ± 27	100 ± 12	105 ± 16	97 ± 18	NS
α2-AR	100 ± 4	89.0 ± 3.1	72.9 ± 7.7*^b^*	75.6 ± 10.3	S
β3-AR	100 ± 10	94.5 ± 5.7	110 ± 9	84.3 ± 5.5	NS
α2/β3- AR	1.03 ± 0.08	0.98 ± 0.07	0.81 ± 0.15	0.91 ± 0.11	NS

Values are expressed in arbitrary units (AU). Mean values of control male rat were set as 100%. Values are expressed as the mean ± S.E.M. of 7 animals per group. ANOVA (p < 0.05): S, sex effect; D, diet effect; SxD, interactive effect; NS, non significant; Student's t-test (p < 0.05): *^a^*HF *vs *Control, *^b^*Female *vs *Male.

Inguinal depot of control female rats showed higher levels of LPL and lower levels of α2-AR and CPT1 than males. HF diet feeding increased LPL but decreased CPT1 levels in male rats. LPL content decreased in HF female rats. HSL, β3-AR, PKA and α2-AR/β3-AR ratio did not change either between sexes or in response to HF diet.

### Correlations between fatty acid content in adipose tissue depots and fatty acid intake or adiposity index

**Figure 1:**
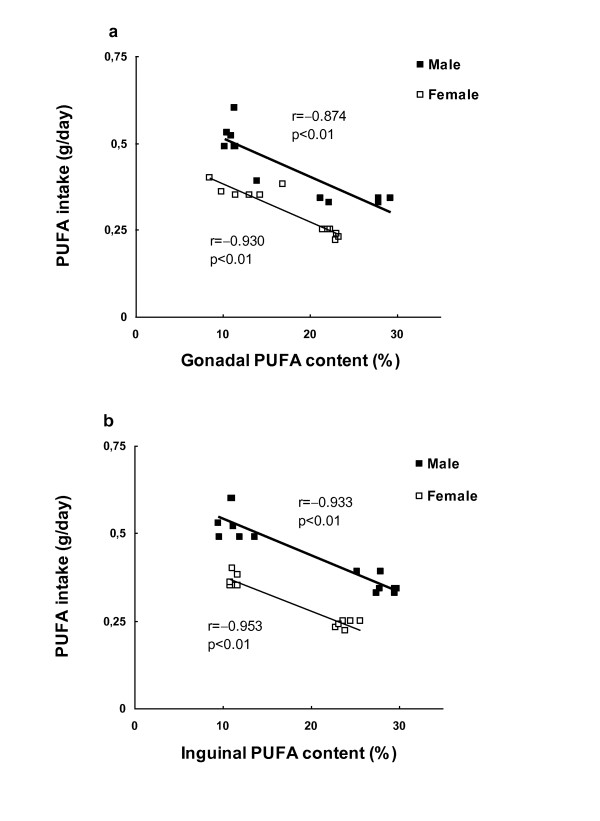
**Correlations between PUFA intake and adipose tissue PUFA depot content**. Gonadal and inguinal PUFA content is expressed as a percentage with respect to the total fatty acids. Pearson's correlation coefficients (r values) and significance levels are shown.

In both adipose tissues, PUFA content negatively correlated with its dietary intake when all the experimental animals were included (Figure 1). Correlations between adipose tissue fatty acid content and adiposity index were very similar in both fat depots (Table 8). MUFA and PUFA content were positively and negatively correlated, respectively, with adiposity index in male rats, but not in females. No significant correlations were obtained between SFA tissue content and adiposity index in either sex.

**Table 8:** Pearson's correlation coefficients between fatty acid content of adipose tissue depots and adiposity index

Adipose tissue fatty acid content	Adiposity index	
		
	Male	Female
Gonadal fat		
SFA	NS	NS
MUFA	0.771**	NS
PUFA	-0.858**	NS
Inguinal fat		
SFA	NS	NS
MUFA	0.791**	NS
PUFA	-0.797**	NS

Fatty acid content was expressed as the percentage of its integrated chromatogram peak area with respect to the total area of fatty acids that were identified (C14:0, C16:0, C18:0, C20:0, C16:1n-7, C18:1n-9, C18:2n-6, C18:3n-3). Adiposity index was calculated as the sum of the weight of lumbar, gonadal, inguinal and mesenteric fat depots expressed as percentage with respect to body weight. Pearson's correlation coefficients (r values) and significance levels are shown. *p < 0.05. **p < 0.01. NS, non significant.

## Discussion

In the literature, very few studies focus on both sex- and diet-dependent differences in rat adipose tissue fatty acid composition and on levels of proteins involved in fatty acid uptake and release.

Diet composition controls nutrient partitioning to adipose tissue depots, especially fatty acids [25,26]. In animals fed with the control diet, the fatty acid proportion in both adipose tissue depots is quite different from that in HF diet, with fat depots showing lower percentages of PUFA and greater proportions of both SFA and MUFA. The accumulation of MUFA and SFA in adipose depots could be related with an enhancement of lipogenic activity derived from increased fatty acid endogenous synthesis from dietary carbohydrates [27-29]. The lower proportion of PUFA in fat depots may be a result of their utilization as precursors of other essential fatty acids and derivates [27,30] and also of their preferential release and low re-uptake [30]. In addition, ΣSFA/ΣMUFA and ΣSFA/ΣPUFA ratios are lower in control diet compared to adipose tissues, which reinforces the idea of active management and/or storage of dietary fatty acids in both fat depots of control animals.

In contrast to a control situation, the proportion of fatty acids in both adipose depots of HF animals is very similar to that of the HF diet, which could be related to the greater fat availability that the dietary treatment implies. This would lead to maintenance in the tissue of the same fatty acid dietary proportion, because of the preferential storage in adipose tissues of the excess dietary fatty acids, despite their active utilization by adipose tissues. Thus, the relative composition of the HF diet is directly reflected in the relative fatty acid composition of adipose tissues as has been previously reported [24,30]. Interestingly, when a correlation analysis, including all experimental animals, was performed between fatty acid intake and fatty acid tissue content, a negative association for PUFA was found, which points to increased PUFA utilization in fat depots when PUFA intake is increased. Moreover, our results also point to a depot dependent response of PUFA content to changes in dietary PUFA, as the lower dispersion of these data in the inguinal depot indicates (see cluster distribution of the data in Figure 1B). Thus, the inguinal depot would be more sensitive to changes in diet composition.

Besides, fatty acid desaturation indexes (16:1/16:0 and 18:1/18:0) have been proposed as indicators of stearoyl-CoA desaturase1 (SCD1) activity, the rate limiting enzyme involved in the biosynthesis of MUFA [31,32] and one of the crucial factors in body fat accumulation [32]. In our study, the decreased desaturation indexes in response to HF diet feeding in both sexes and in both depots could be related with a lower synthesis of MUFA from SFA. In addition, the lower carbohydrate intake and greater fatty acid availability of HF animals compared to controls also suggests a lower lipogenic activity in both adipose depots. In fact, it has been reported that SCD1 activity is elevated by dietary carbohydrate, and mice lacking SCD1 are characterized by decreased fatty acid synthesis and increased fatty acid oxidation [31]. Thus, the diet-induced decrease of the desaturation index observed in this study could be understood as a mechanism to protect the animal from the development of obesity brought on by HF diet feeding. Interestingly, the reduction of 18:1/18:0 ratio in inguinal fat of HF male rats was lower than in females, which could be associated to the greater tissue weight gain shown by males in response to diet. In this sense, the decrease of CPT1 levels in both depots of HF males would reflect lower fatty acid oxidation and would contribute to the sexual dimorphism found in body fat accumulation in response to the HF diet.

Visceral fat accumulation could be considered a risk factor for metabolic syndrome [2]. However, the high proportion of 18:1n-9 in both diets and adipose tissue depots might play a protective role against cardiovascular diseases [27,33]. Moreover, the reduction of the desaturation index observed in response to HF diet feeding would prevent inflammation in white adipose depots, as previously reported in diet-induced obesity mouse models [34]. In fact, in a previous study using this same experimental model, we reported that this HF diet did not entail either a more pro-inflammatory, pro-atherogenic adipokine or lipid serum profile [35].

The extent to which obesity is induced by diet varies depending on the length of the feeding period and the quality and quantity of dietary fat and the proportion of the other macronutrients in the diet [13,36]. In the present study we used an unbalanced high-fat diet with a lower carbohydrate content and modified fatty acid composition in comparison with a control diet, which does not induce body weight gain, but promotes body fat accumulation that was sex dependent. The higher adiposity in HF males could be related to their low energy expenditure and whole body oxygen consumption, which we have previously described in the same experimental model [37]. In addition, only male rats show a significant negative correlation between adipose PUFA content and adiposity index in both adipose tissues, which suggests the existence of sex differences in the adipogenic effect of PUFA depending on their dietary content [38]. In this sense, we also found a positive correlation between PUFA intake and adiposity index for male rats (r = 0.881, p < 0.05) but not for females. All in all, our findings are in agreement with the idea of a sex dependent effect of dietary fatty acids on body fat accumulation.

The great body fat accumulation in HF male rats could also be due to sexual differences in the effect of HF diet on serum sex hormones. In fact, HF diets reduce serum testosterone levels [39], which has been associated to increased intra-abdominal fat [40]. However, in female rats, estrogen levels increase in response to HF diet [41], which would prevent variations of adiposity index in this sex. Thus, exogenous administration of estrogens to ovariectomized rats has been reported to limit the increase of adiposity [42].

The changes induced by HF diet feeding on the levels of adipose tissue proteins involved in lipid uptake and mobilization are also both depot- and sex-dependent. Thus, the sex differences observed in inguinal depot weight gain could be attributed, in part, to the differences in LPL levels, which increased in HF male rats and decreased in HF females. Accordingly, a positive correlation between inguinal LPL levels and adiposity index was observed only in male rats (r = 0.631, p < 0.01). In addition, in this tissue, male rats also showed slightly higher levels of the antilypolitic α2-AR, which could also contribute to the greater increase of the size of this depot.

In contrast, the sex dependent changes observed in the gonadal depot weight could be related with differences in lipolytic capacity. In female rats, HF diet induced an increase of proteins involved in the adrenergic control of lipolytic pathway, with a higher α2/β3-AR ratio (Table 7) and a lower LPL/HSL ratio (0.62 ± 0.15 for HF females and 1.75 ± 0.39 for HF males, p < 0.05), contributing to a lesser accumulation of fat in the visceral region of female rats. Although HF male rats also exhibited an increase in the main proteins mediating lipolysis, this effect does not seem to be enough to limit the fat accumulation in the gonadal depot due, in part, to the decreased CPT1 levels, which would compromise the oxidation of fatty acids by promoting their accumulation.

In summary, the HF diet used in this study induced a sex- and depot-dependent increase in adiposity without changes in body weight. The fatty acid content of the HF diet was reflected in the fatty acid tissue composition, as it induced an increase in the proportion of MUFA and a decrease in that of PUFA in both fat depots. The differences in adiposity index between male and female rats reflect the differential sex response to dietary fatty acid content and could be related to the levels of the proteins involved in tissue lipid handling. In male rats, fat accumulation in inguinal tissue is associated to an increased ability for fatty acid uptake, whereas in gonadal tissue it is related to impaired fatty acid oxidation.

## Abbreviations

AR: adrenergic receptor; CPT1: carnitine palmitoyltransferase 1; HF: high-fat; HSL: hormone sensitive lipase; LPL: lipoprotein lipase; MUFA: monounsaturated fatty acid; PKA: protein kinase A; PPAR: peroxisome proliferator-activated receptor; PUFA: polyunsaturated fatty acid; SCD1: stearoyl-CoA desaturase 1; SFA: saturated fatty acid.

## Competing interests

The authors declare that they have no competing interests.

## Authors' contributions

MEE performed all the experiments, analyzed the data and participated in writing the manuscript. AMP participated in the experimental design and in the discussion of the results. IL and MG designed, supervised and coordinated the study and prepared the manuscript. All authors revised the manuscript and approved the final version.

## References

[B1] Trayhurn P Endocrine and signalling role of adipose tissue: new perspectives on fat Acta Physiol Scand 2005 184 285 293 10.1111/j.1365-201X.2005.01468.x 16026420

[B2] Atzmon G Yang XM Muzumdar R Ma XH Gabriely I Barzilai N Differential gene expression between visceral and subcutaneous fat depots Horm Metab Res 2002 34 622 628 10.1055/s-2002-38250 12660871

[B3] Hernandez-Morante JJ Perez-de-Heredia F Lujan JA Zamora S Garaulet M Role of DHEA-S on body fat distribution: gender- and depot-specific stimulation of adipose tissue lipolysis Steroids 2008 73 209 215 10.1016/j.steroids.2007.10.005 18063002

[B4] Perrini S Laviola L Cignarelli A Melchiorre M De Stefano F Caccioppoli C Natalicchio A Orlando MR Garruti G De Fazio M Catalano G Memeo V Giorgino R Giorgino F Fat depot-related differences in gene expression, adiponectin secretion, and insulin action and signalling in human adipocytes differentiated in vitro from precursor stromal cells Diabetologia 2008 51 155 164 10.1007/s00125-007-0841-7 17960360

[B5] Williams CM Lipid metabolism in women Proc Nutr Soc 2004 63 153 160 10.1079/PNS2003314 15070445

[B6] Mead JR Irvine SA Ramji DP Lipoprotein lipase: structure, function, regulation, and role in disease J Mol Med 2002 80 753 769 10.1007/s00109-002-0384-9 12483461

[B7] Carey GB Mechanisms regulating adipocyte lipolysis Adv Exp Med Biol 1998 441 157 170 9781323 10.1007/978-1-4899-1928-1_15

[B8] McGarry JD Brown NF The mitochondrial carnitine palmitoyltransferase system. From concept to molecular analysis Eur J Biochem 1997 244 1 14 10.1111/j.1432-1033.1997.00001.x 9063439

[B9] Voshol PJ Rensen PC van Dijk KW Romijn JA Havekes LM Effect of plasma triglyceride metabolism on lipid storage in adipose tissue: studies using genetically engineered mouse models Biochim Biophys Acta 2009 1791 479 485 19168150 10.1016/j.bbalip.2008.12.015

[B10] Pujol E Rodriguez-Cuenca S Frontera M Justo R Llado I Kraemer FB Gianotti M Roca P Gender- and site-related effects on lipolytic capacity of rat white adipose tissue Cell Mol Life Sci 2003 60 1982 1989 10.1007/s00018-003-3125-5 14523558 PMC11138756

[B11] Llado I Estrany ME Rodriguez E Amengual B Roca P Palou A Effects of cafeteria diet feeding on beta3-adrenoceptor expression and lipolytic activity in white adipose tissue of male and female rats Int J Obes Relat Metab Disord 2000 24 1396 1404 10.1038/sj.ijo.0801390 11126334

[B12] Imbeault P Almeras N Richard D Despres JP Tremblay A Mauriege P Effect of a moderate weight loss on adipose tissue lipoprotein lipase activity and expression: existence of sexual variation and regional differences Int J Obes Relat Metab Disord 1999 23 957 965 10.1038/sj.ijo.0801025 10490802

[B13] Petzke KJ Riese C Klaus S Short-term, increasing dietary protein and fat moderately affect energy expenditure, substrate oxidation and uncoupling protein gene expression in rats J Nutr Biochem 2007 18 400 407 10.1016/j.jnutbio.2006.07.005 16979329

[B14] Mann JI Diet and risk of coronary heart disease and type 2 diabetes Lancet 2002 360 783 789 10.1016/S0140-6736(02)09901-4 12241840

[B15] Grundy SM The optimal ratio of fat-to-carbohydrate in the diet Annu Rev Nutr 1999 19 325 341 10.1146/annurev.nutr.19.1.325 10448527

[B16] de Heredia FP Larque E Zamora S Garaulet M Dehydroepiandrosterone modifies rat fatty acid composition of serum and different adipose tissue depots and lowers serum insulin levels J Endocrinol 2009 201 67 74 10.1677/JOE-08-0432 19144736

[B17] Jump DB Clarke SD Thelen A Liimatta M Coordinate regulation of glycolytic and lipogenic gene expression by polyunsaturated fatty acids J Lipid Res 1994 35 1076 1084 8077846

[B18] Clarke SD Polyunsaturated fatty acid regulation of gene transcription: a molecular mechanism to improve the metabolic syndrome J Nutr 2001 131 1129 1132 11285313 10.1093/jn/131.4.1129

[B19] Llado I Pons A Palou A Effects of fasting on lipoprotein lipase activity in different depots of white and brown adipose tissues in diet-induced overweight rats J Nutr Biochem 1999 10 609 614 10.1016/S0955-2863(99)00050-9 15539256

[B20] Lowry OH Rosebrough NJ Farr AL Randall RJ Protein measurement with the Folin phenol reagent J Biol Chem 1951 193 265 275 14907713

[B21] Thomas PS Farquhar MN Specific measurement of DNA in nuclei and nucleic acids using diaminobenzoic acid Anal Biochem 1978 89 35 44 10.1016/0003-2697(78)90724-8 707807

[B22] Folch J Lees M Sloane Stanley GH A simple method for the isolation and purification of total lipides from animal tissues J Biol Chem 1957 226 497 509 13428781

[B23] Peuchant E Wolff R Salles C Jensen R One-step extraction of human erythrocyte lipids allowing rapid determination of fatty acid composition Anal Biochem 1989 181 341 344 10.1016/0003-2697(89)90254-6 2817399

[B24] Llado I Pons A Palou A Changes in fatty acid composition in rat adipose tissue induced by dietary obesity Biochem Mol Biol Int 1996 40 295 303 8896751 10.1080/15216549600201782

[B25] Lewis GF Carpentier A Adeli K Giacca A Disordered fat storage and mobilization in the pathogenesis of insulin resistance and type 2 diabetes Endocr Rev 2002 23 201 229 10.1210/er.23.2.201 11943743

[B26] Paniagua JA Gallego de la Sacristana A Romero I Vidal-Puig A Latre JM Sanchez E Perez-Martinez P Lopez-Miranda J Perez-Jimenez F Monounsaturated fat-rich diet prevents central body fat distribution and decreases postprandial adiponectin expression induced by a carbohydrate-rich diet in insulin-resistant subjects Diabetes Care 2007 30 1717 1723 10.2337/dc06-2220 17384344

[B27] Garaulet M Perez-Llamas F Perez-Ayala M Martinez P de Medina FS Tebar FJ Zamora S Site-specific differences in the fatty acid composition of abdominal adipose tissue in an obese population from a Mediterranean area: relation with dietary fatty acids, plasma lipid profile, serum insulin, and central obesity Am J Clin Nutr 2001 74 585 591 11684525 10.1093/ajcn/74.5.585

[B28] Pichon L Huneau JF Fromentin G Tome D A high-protein, high-fat, carbohydrate-free diet reduces energy intake, hepatic lipogenesis, and adiposity in rats J Nutr 2006 136 1256 1260 16614413 10.1093/jn/136.5.1256

[B29] Shankar K Harrell A Kang P Singhal R Ronis MJ Badger TM Carbohydrate-Responsive Gene Expression in the Adipose Tissue of Rats Endocrinology 2009 19880807 10.1210/en.2009-0840

[B30] Raclot T Selective mobilization of fatty acids from adipose tissue triacylglycerols Prog Lipid Res 2003 42 257 288 10.1016/S0163-7827(02)00066-8 12689620

[B31] Flowers MT Ntambi JM Role of stearoyl-coenzyme A desaturase in regulating lipid metabolism Curr Opin Lipidol 2008 19 248 256 10.1097/MOL.0b013e3282f9b54d 18460915 PMC4201499

[B32] Jeyakumar SM Lopamudra P Padmini S Balakrishna N Giridharan NV Vajreswari A Fatty acid desaturation index correlates with body mass and adiposity indices of obesity in Wistar NIN obese mutant rat strains WNIN/Ob and WNIN/GR-Ob Nutr Metab (Lond) 2009 6 27 10.1186/1743-7075-6-27 19519902 PMC2704216

[B33] Berry EM Eisenberg S Haratz D Friedlander Y Norman Y Kaufmann NA Stein Y Effects of diets rich in monounsaturated fatty acids on plasma lipoproteins--the Jerusalem Nutrition Study: high MUFAs vs high PUFAs Am J Clin Nutr 1991 53 899 907 2008870 10.1093/ajcn/53.4.899

[B34] Liu X Miyazaki M Flowers MT Sampath H Zhao M Chu K Paton CM Joo DS Ntambi JM Loss of Stearoyl-CoA desaturase-1 attenuates adipocyte inflammation: effects of adipocyte-derived oleate Arterioscler Thromb Vasc Biol 30 31 38 10.1161/ATVBAHA.109.195636 19910642 PMC2837593

[B35] Thomas-Moya E Gianotti M Proenza AM Llado I Paraoxonase 1 response to a high-fat diet: gender differences in the factors involved Mol Med 2007 13 203 209 10.2119/2006-00078.Thomas-Moya 17592556 PMC1892762

[B36] Archer ZA Rayner DV Rozman J Klingenspor M Mercer JG Normal distribution of body weight gain in male Sprague-Dawley rats fed a high-energy diet Obes Res 2003 11 1376 1383 10.1038/oby.2003.186 14627759

[B37] Catala-Niell A Estrany ME Proenza AM Gianotti M Llado I Skeletal muscle and liver oxidative metabolism in response to a voluntary isocaloric intake of a high fat diet in male and female rats Cell Physiol Biochem 2008 22 327 336 10.1159/000149811 18769060

[B38] de Heredia FP Larque E Del Puy Portillo M Canteras M Zamora S Garaulet M Age-related changes in fatty acids from different adipose depots in rat and their association with adiposity and insulin Nutrition 2008 24 1013 1022 10.1016/j.nut.2008.03.022 18562171

[B39] Cano P Jimenez-Ortega V Larrad A Reyes Toso CF Cardinali DP Esquifino AI Effect of a high-fat diet on 24-h pattern of circulating levels of prolactin, luteinizing hormone, testosterone, corticosterone, thyroid-stimulating hormone and glucose, and pineal melatonin content, in rats Endocrine 2008 33 118 125 10.1007/s12020-008-9066-x 18449810

[B40] El Hafidi M Perez I Carrillo S Cardoso G Zamora J Chavira R Banos G Effect of sex hormones on non-esterified fatty acids, intra-abdominal fat accumulation, and hypertension induced by sucrose diet in male rats Clin Exp Hypertens 2006 28 669 681 10.1080/10641960601013617 17132534

[B41] Cifuentes M Morano AB Chowdhury HA Shapses SA Energy restriction reduces fractional calcium absorption in mature obese and lean rats J Nutr 2002 132 2660 2666 12221226 10.1093/jn/132.9.2660 PMC4010555

[B42] Shinoda M Latour MG Lavoie JM Effects of physical training on body composition and organ weights in ovariectomized and hyperestrogenic rats Int J Obes Relat Metab Disord 2002 26 335 343 10.1038/sj.ijo.0801900 11896488

